# Non-circadian aspects of BHLHE40 cellular function in cancer

**DOI:** 10.18632/genesandcancer.201

**Published:** 2020

**Authors:** Zsofia Kiss, Maria Mudryj, Paramita M. Ghosh

**Affiliations:** ^1^ VA Northern California Health Care System, Sacramento, CA, USA; ^1^ Department of Urology, University of California Davis School of Medicine, Sacramento, CA, USA; ^1^ Department of Biochemistry and Molecular Medicine, University of California Davis School of Medicine, Sacramento, CA, USA; ^1^ Department of Microbiology and Immunology, University of California, Davis, CA, USA

**Keywords:** BHLHE40/DEC1/STRA13/SHARP2, E-box, PI3K/Akt/mTOR, AMPK, HIF-1α

## Abstract

While many genes specifically act as oncogenes or tumor suppressors, others are tumor promoters or suppressors in a context-dependent manner. Here we will review the basic-helix-loop-helix (BHLH) protein BHLHE40, (also known as BHLHB2, STRA13, DEC1, or SHARP2) which is overexpressed in gastric, breast, and brain tumors; and downregulated in colorectal, esophageal, pancreatic and lung cancer. As a transcription factor, BHLHE40 is expressed in the nucleus, where it binds to target gene promoters containing the E-box hexanucleotide sequence, but can also be expressed in the cytoplasm, where it stabilizes cyclin E, preventing cyclin E-mediated DNA replication and cell cycle progression. In different organs BHLHE40 regulates different targets; hence may have different impacts on tumorigenesis. BHLHE40 promotes PI3K/Akt/mTOR activation in breast cancer, activating tumor progression, but suppresses STAT1 expression in clear cell carcinoma, triggering tumor suppression. Target specificity likely depends on cooperation with other transcription factors. BHLHE40 is activated in lung and esophageal carcinoma by the tumor suppressor p53 inducing senescence and suppressing tumor growth, but is also activated under hypoxic conditions by HIF-1α in gastric cancer and hepatocellular carcinomas, stimulating tumor progression. Thus, BHLHE40 is a multi-functional protein that mediates the promotion or suppression of cancer in a context dependent manner.

## INTRODUCTION

The study of the biology of cancer identified many oncogenes such as the transcription factor c-myc [1], the regulatory GTP-binding protein Ras [2], and the receptor tyrosine kinase epidermal growth factor receptor (EGFR) [3]. In cancers, these oncogenes either are overexpressed or experience gain-of-function mutations. In addition, multiple tumor suppressors have also been identified, which either are deleted or experience a loss of function mutation in cancer. This includes the retinoblastoma protein (RB) [4], the p53 tumor suppressor protein [5] and phosphatase and tensin homolog (PTEN) [6]. Restoration of wildtype tumor suppressors mostly prevents tumor progression and even induces tumor regression.

However, cancer literature also describes genes and associated proteins that are upregulated in some cancers and downregulated in others. The regulatory cytokine transforming growth factor β (TGFβ) has tumor suppressive properties and its mis-regulation may result in tumor development or progression [7]. However, TGFβ also regulates metastasis [8], the immune system and the tumor microenvironment [9] to promote tumor progression. Here we will discuss a transcription factor that has a similar dual function - the Class E basic helix-loop-helix protein 40 (BHLHE40), also known as BHLHB2, STRA13, DEC1, or SHARP2.

BHLHE40 is a member of the basic helix-loop-helix (bHLH) protein family, a large superfamily of transcriptional regulators expressed in many organisms. The bHLH superfamily function in a wide variety of physiological processes including control of the cell cycle, regulation of genes associated with the circadian rhythm, differentiation and development of muscle, nervous system, etc. [10-13]. About 118 genes encoding proteins of this family have been identified in humans – many more are identified in yeasts, plants and other organisms. Its name BHLHE40 (also called BHLHB2) comes from its structure [14], while it was named STRA13 because its expression is STimulated by Retinoic Acid [10]. The name SHARP2 came from its close resemblance to the *Drosophila* Split-and-HAiry Related Proteins [15, 16], while *DEC1* is an acronym for Differentiated in Embryonic Chondrocytes [17] (not to be confused with the tumor suppressor gene Deleted in Esophageal Cancer also called DEC1, also called Candidate Tumor Suppressor 9 or CTS9 [18]). To avoid confusion, in this manuscript this gene will be referred to as *BHLHE40*.

**Figure 1:**
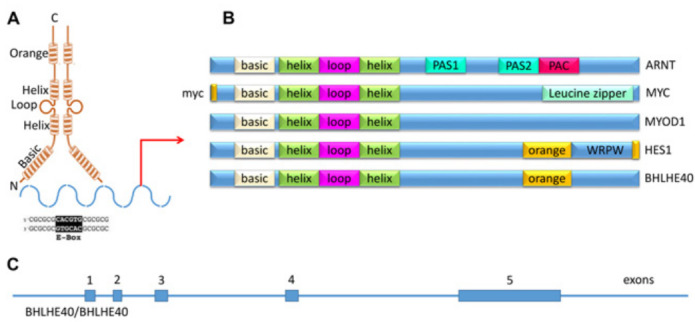
Domains of BHLHE40. **A**. Schematic representing structure of BHLHE40 showing the N terminal end expressing the basic domain that allows DNA binding, the HLH domain that allows the formation of homo/heterodimeric complexes and the orange domain whose function is not yet known. BHLHE40 is shown to bind to an E-box sequence (CACGTG) in the promoters of target genes. **B**. Comparison of the components of members of the bHLH family. All members possess a basic domain and a HLH domain. There are other domains that can be present or absent among the bHLH members. As such, ARNT has two PAS domains required for dimerization with other proteins with PAS domain, and a PAC domain that is necessary for PAS domain folding. In contrast, Myc has a MYC domain that is required for the recruitment of coactivators at the N-terminal, and at the very C terminal end has a leucine-zipper domain for stable dimerization with other leucine-zipper domain containing proteins. It does not express either PAS or PAC. MyoD does not possess other domains besides the basic and HLH domain, while Hes1 expresses an orange domain followed by the tetrapeptide sequence WRPW. The WRPW tetrapeptide is required for Hes1’ suppressor activity. BHLHE40 has also the orange domain, but does not have any tetrapeptide sequence at its C terminus. **C**. Representation of the BHLHE40 gene showing the location of 5 exons.

Members of the bHLH superfamily possess two highly conserved and functionally distinct domains that together comprise a region of about 60 amino-acid residues (reviewed in [19]). The N-terminal end of this region comprises a DNA- binding basic region of 15 amino acids that allows these transcription factors to bind to DNA sequences containing the hexanucleotide E-box (CANNTG) or N-box (CANNAG) sequences [20, 21] (Figure 1A). Classification of these members are based on evolutionary relationships and take into account E-box or N-box binding, conservation of residues in other parts of their motif, and the presence or absence of additional motifs [22] (Table 1). Although BHLHE40 is classified as a Class E protein, it binds a Class B type E-box (CACGTG) [23, 24], due to the presence of a proline at residue 56 and an arginine at residue 58 that enables this binding [25]. At the C-terminal end of this region is the helix-loop-helix (HLH) domain consisting of two α-helices separated by a variable loop region which, along with other domains downstream, allows the transcription factor to form homo/heterodimeric complexes [21, 26] (Figure 1A). Evolutionary classification of the bHLH transcription factors also relies on the presence or absence of additional domains at the C-terminal end. This includes PAS (PER, ARNT, SIM) domains that function as dimerization motifs [27], PAC (C-terminal from the PAS domain), responsible for PAS domain folding, orange domain, which include hairy-related proteins such as BHLHE40 and BHLHE41 [28] and leucine-zipper domains (Figure 1B). The molecular function of the orange domain is not precisely known, although it has been proposed that it mediates target specificity and transcriptional repression (as well as binding partners) [29]. There is also evidence that both orange and leucine-zipper domains mediate dimerization [28, 1].

The human *BHLHE40* gene is located on chromosome 3p26.1, spanning ~2.4 kb, and containing 5 exons (Figure 1C). Structurally BHLHE40 possesses a bHLH domain close to the N-terminal region of the protein. It contains an Orange domain but does not possess a PAS domain (Figure 1A). BHLHE40 can homodimerize (Figure 1A), but often heterodimerizes with the related BHLHE41, with which it shares 97% homology in the bHLH domain (Asp vs Glu at the N-terminal residue) and 52% homology in the orange domain [30]. BHLHE40 is expressed in a wide range of human tissues, and interacts with numerous nuclear proteins [31-34]. Apart from E-box binding, BHLHE40 was also shown to suppress transcription by binding to SP1 domains on target genes [35]. Targets of BHLHE40 regulated transcription are listed in Table 2.

BHLHE40 is regulated by a number of important signaling pathways and transcription factors, such as TGFβ, hypoxia inducible factor (HIF), CLOCK-BMAL1 heterodimers and RORα [36-39]. It has been implicated in multiple cellular functions, including chondrocyte differentiation [12], regulation of circadian rhythmicity [13, 40-42], memory CD8+ T cell development [43], organ rejection following transplantation [44, 45], skeletal muscle regeneration [46, 47], adipogenesis [39, 48-51], neurogenesis [10. 52], and in regulation of hypoxia [53-55]. Numerous studies have shown BHLHE40 responds to stress stimuli, such as DNA damage [56] and that its expression increases due to ionizing radiation in a p53-independent manner but can regulate the amount of p53 through direct interaction with the molecule [57]. In this article, we will investigate which cellular functions of BHLHE40 are involved in the differential effects of this transcription factor in different types of cancers.

## EXPRESSION OF BHLHE40 IN CANCER

**Figure 2:**
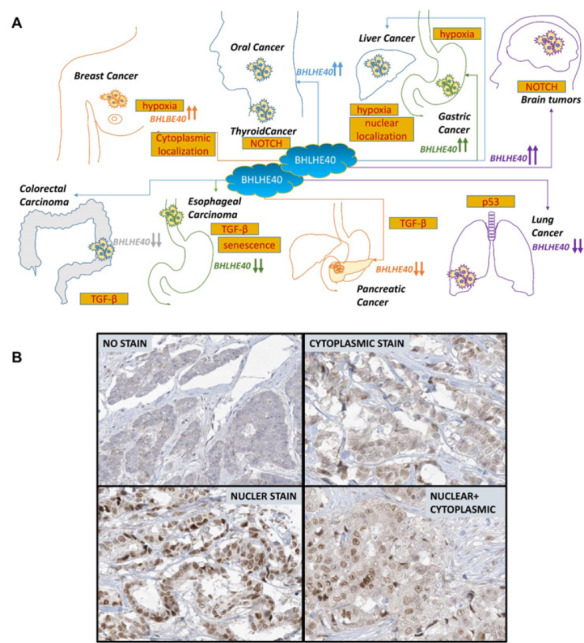
Differential expression and subcellular localization of BHLHE40 in various cancers. **A**. BHLHE40 is upregulated in some cancers and downregulated in others. Compared to non-tumor controls, BHLHE40 expression is increased in thyroid, gastric, breast and brain tumors whereas it is downregulated in colorectal, esophageal, pancreatic and non-small cell lung cancer. In hepatocellular and oral squamous cell carcinoma, BHLHE40 is increased in tumors compared to normal cells, but decreases from well-differentiated to poorly differentiated tumors. Proposed mechanisms of these effects as described in the literature are also shown. B. Immunohistochemical photomicrographs of BHLHE40 in various cancer showing (i) no BHLHE40 staining, (ii) mostly cytoplasmic BHLHE40 staining, (iii) mostly nuclear BHLHE40 staining and (iv) both nuclear and cytoplasmic BHLHE40 staining. Courtesy: The Human Protein Atlas (https://www.proteinatlas.org).

The expression patterns of BHLHE40 and its impact on tumor development are tumor type-specific - it is suppressed in some types of cancer and overexpressed in others [17, 58-60] (Figure 2A). Moreover, in some tumors, BHLHE40 appears to have a bimodal function – it is upregulated during tumor initiation, whereas its expression is lost during tumor progression, exhibiting a significant decrease in expression from well-differentiated to poorly differentiated tumors. BHLHE40 is primarily a transcriptional regulator [28, 61, 37] that is often deleted or downregulated in cancer, including Burkitt’s lymphoma [62], osteosarcoma [63], non-small cell lung cancer [64] and in pancreatic cancer [65]. Paradoxically, BHLHE40 is also upregulated in many cancers, such as in gastric cancer [66, 67] and in breast cancer [68]. Here we will discuss the different conditions where it is upregulated or downregulated.

### Cancers where BHLHE40 expression is upregulated

#### Breast cancer

At least 5 individual studies have indicated that *BHLHE40* is upregulated in breast cancer tissue. Investigations of 253 breast cancer patients demonstrated an increase in BHLHE40 expression from normal to in situ as well as invasive breast carcinoma [68]. Elevated expression of BHLHE40 was observed in endothelial, fibroblasts and inflammatory cells of the patients in addition to tumor cells. BHLHE40 expression correlated positively with tumor grade (*p* = 0.01), and with various angiogenic factors [68]. In two studies of about 1200 breast cancer patients each, BHLHE40 was one of multiple markers predicting disease outcome and metastatic risk [69, 70]. A fourth study of 1080 patients with primary invasive ductal carcinoma showed that BHLHE40 expression increased from normal to benign to premalignant and plateaued from premalignant to malignant phenotype [71]. Another study of 147 patients with invasive breast ductal carcinomas showed that BHLHE40 expression was elevated in invasive ductal carcinomas and positively correlated with tumor grade (*P* = 0.023) [72]. Thus, BHLHE40 is consistently upregulated in breast cancer, irrespective of the subtype of breast cancer investigated.

**Table 1:** Phylogenetic classification of bHLH proteins based on binding sequence to target DNA, and the presence or absence of additional motifs

Phylogenetic Group	Binding Sequence	Structural Characteristics	Proteins in class
Class A	CAGCTG (E-box), CACCTG (E-box)		MyoD, Neurogenin, E12/E47, NeuroD, Atonal, Mist, Beta3, Oligo, Net, Mesp, Twist, Paraxis, MyoR, Hand, PTFa/b, SCL, NSCL
Class B	CACGTG (E-box), CATGTTG (E-box)		SRC, Figa, Myc, MAD, Mnt, Max, USF, MITF, SREBP, AP4, MLX, TF4
Class C	ACGTG, GCGTG	PAS domain	Clock, ARNT, Bmal, AHR, Sim, Trh, HIF
Class D	-		Emc
Class E	CACGCG, CACGAG (N-box)	Orange domain, WRPW peptide	Hey, Hairy, E(Spl)
Hybrid class E/B	CACGTG (E-box) (Class B)	Orange domain (Class E)	BHLHE40, BHLHE41
Class F		COE domain	COE

The N-terminal end of BHLH proteins comprises a DNA- binding basic region of 15 amino acids that allows these transcription factors to bind to DNA sequences containing the hexanucleotide E-box (CANNTG) or N-box (CANNAG) sequences. BHLHE40 and BHLHE41 belong to a hybrid class since they contain an Orange domain like class E proteins but bind to the canonical CACGTG E-box sequence similar to class B proteins. The following information is based on Table 1 of Jones, Genome Biol. 2004; 5(6): 226.

#### Brain tumors

Many different types of brain cancers have been characterized, and in almost all of them, BHLHE40 expression has been shown to be higher than in surrounding non-tumor tissue. BHLHE40 was upregulated in glioma compared to non-tumor brain tissue and played an oncogenic role in glioma cells [73]. Immunohistochemical analysis of 157 patients with newly diagnosed glioma and 63 with recurrent glioblastoma who relapsed during temozolomide (TMZ) chemotherapy showed that high BHLHE40 expression was significantly associated with high pathological tumor grade and poor response to TMZ [74]. In patients with recurrent glioblastoma, BHLHE40 expression also correlated negatively with apoptosis [74]. In another study, which analyzed 44 primary and 16 recurrent oligodendroglial neoplasms with 1p-aberrations, high BHLHE40 expression was observed in the cell nuclei in almost all (56 of 60) tumors, and occasionally in endothelial cells, as well as in glial and neuronal cells of surrounding brain tissue, compared to those away from the tumor [75].

Meningiomas are primary brain or spinal cord tumors that are aggressive in only a minority of cases. Grade I meningiomas are not aggressive but Grade II malignancies are known to infiltrate the surrounding brain tissue. A study that classified these tumors according to the expression of the tumor suppressor gene *deleted in colorectal cancer (DCC)*, whose loss marks the aggressiveness of the meningiomas, showed that the expression of BHLHE40 was significantly upregulated in the DCC^low^ (highly aggressive) tumors [76]. Taken together, these studies demonstrate that in all subtypes of brain carcinoma, tumor aggression is associated with an increase in the expression of BHLHE40.

**Table 2:** Selected confirmed targets of BHLHE40

Gene Name	Gene Symbol	Effect of BHLHE40 on transcription	Co-transcription factor
Clusterin	CLU	upregulation	SP1
Snail Family Transcriptional Repressor 1	SNAI1	Repression	SP1
Snail Family Transcriptional Repressor 2	SNAI2	Repression	SP1
Twist Family BHLH Transcription Factor 1	TWIST1	Repression	SP1
Interleukin 10	IL-10	Repression	
Signal Transducer And Activator Of Transcription 1	STAT1	Repression	VHL
Fas Cell Surface Death Receptor	FAS	upregulation	STAT3
Survivin (baculoviral inhibitor of apoptosis repeat-containing 5)	BIRC5	upregulation	SP1
Jagged1	JAG1	upregulation	Notch1
Cyclin D1	CCND1	Repression	SUMO, HDAC1
Macrophage Inhibitory Cytokine 1/ Growth Differentiation Factor 15	MIC1/GDF15	repression	TP53
Phosphatidylinositol-4,5-Bisphosphate 3-Kinase Catalytic Subunit Alpha	PIK3CA	upregulation	SP1/β-catenin
Period circadian protein homolog 1	PER1	repression	
Peroxisome Proliferator-Activated Receptor Gamma Coactivator 1-Alpha	PGC-1α	Repression	histone deacetylases (HDACs)
runt-related transcription factor 2	RUNX2	Upregulation	SP1
Alkaline Phosphatase	ALP	upregulation	SP1
β-catenin	CTNNB1	upregulation	SP1

15617 target genes of the BHLHE40 transcription factor have been identified in ChIP-seq datasets from the ENCODE Transcription Factor Targets dataset. However, of these only a few have been verified in laboratory experiments and confirmed to be direct BHLHE40 targets. Below we present a partial list of BHLHE40 transcriptional targets that have been identified over the past several years.

#### Gastric cancer

BHLHE40 was upregulated in gastric cancer compared with normal tissue. Two studies showed that 83% gastric cancer tissues stained positive for BHLHE40 [66, 67] and expression increased during the tumor progression from well differentiated to poorly differentiated [66, 67]. In contrast, weak staining for BHLHE40 was observed in 10-23% normal tissues (1/10) [66, 67]. Thus, BHLHE40 appears to be significantly upregulated in gastric cancer and associated with tumor differentiation status.

#### Thyroid cancer

In a retrospective cohort of 54 thyroid cancers, the large majority of malignant lesions (92.5%) displayed a strong expression of BHLHE40 specifically in tumor cells, while normal adjacent thyroid tissue did not express BHLHE40 [77]. Significantly, BHLHE40 was observed in both well-differentiated and undifferentiated thyroid cancer, indicating that is unlikely to have a role in the differentiation status of the tumor [77].

### Cancers where BHLHE40 expression is downregulated:

#### Colorectal cancer

While BHLHE40 has been positively associated with tumorigenesis in certain malignancies, in other cellular contexts this association is negative. A significant example of this negative association is in colorectal carcinoma cells, where loss of BHLHE40 correlated with a high proliferation index, whereas BHLHE40 overexpression correlated with a low mitotic index [78]. While BHLHE40 levels were increased in colon cancer tissue compared to normal colon, cell cycle blockers markedly induced BHLHE40 expression. Moreover, in cell culture studies, BHLHE40 overexpression inhibited proliferation, impeded serum deprivation-induced apoptosis and selectively inhibited the activation of procaspases [79]. Subsequent studies described an alternate means by which BHLHE40 may suppress the growth of colorectal carcinoma. BHLHE40 expressing BHLHE40^+^ T_H_1-like immunoregulatory cells were enriched in tumors with microsatellite-insatiability and these tumors who are responsive to immune-checkpoint blockade [80]. Therefore, BHLHE40 expression has a tumor suppressive effect in colorectal cancer.

**Figure 3:**
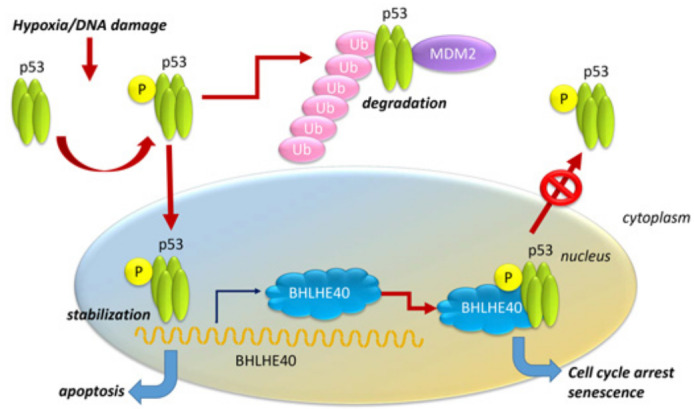
Regulation of BHLHE40 by the p53-dependent pathway in senescence. BHLHE40 can be regulated by the DNA-damage associated gene p53, a tumor suppressor, which is commonly mutated in several cancers resulting in its loss of function, thus allowing uncontrolled cell growth and proliferation in affected tumors. TP53 is activated in a tetramer form and is expressed normally in low levels through interaction with mouse double mutant 2 (Mdm2) which signals for its degradation via ubiquitination (“Ub”). Under conditions of stress, including DNA damage, p53 gets activated and is stabilized by dissociation from Mdm2, and is transported to the nucleus, where it regulates the transcriptional activity of several target genes, including BHLHE40. Binding of BHLHE40 to p53 stabilizes the complex in the nucleus and allows cooperative transcription of target genes that may lead to senescence or apoptosis.

#### Pancreatic carcinoma

A strong association has been made in pancreatic cancer between nuclear BHLHE40 and tumor suppression. BHLHE40 expression analyses were carried out in normal pancreas (*n* = 10), pancreatic ductal adenocarcinoma (*n* = 77), and in eight pancreatic cancer cell lines [65]. Patients with weak/absent nuclear BHLHE40 staining had significantly worse median survival compared to those with strong staining (13 months vs. 27 months, *P* = 0.03) [65]. Other studies, however, showed increased *cytoplasmic* BHLHE40 in pancreatic cancer –supporting a tumor suppressive role for nuclear BHLHE40. In this study, higher levels of BHLHE40 was observed in pancreatic carcinoma compared to low levels in non-tumor tissue; however, it was noted that the increased BHLHE40 expression was confined to the cytoplasm, where it’s transcription activity would be suppressed [81]. Analysis of the Cancer Genome Atlas (TCGA) also showed that low expression of BHLHE40 was associated with favorable prognosis in pancreatic cancer patients [82]. Together, these reports suggest a tumor suppressive role of nuclear BHLHE40 in pancreatic cancer.

#### Non-small cell lung carcinoma

In a retrospective study focusing on archived non-small-cell lung carcinoma (NSCLC) tissues, investigators found that in 118 patient samples, BHLHE40 expression is markedly reduced (30.5% positivity) in cancer samples when compared with adjacent normal lung tissues (89.8%) [64]. Loss of BHLHE40 was correlated with poor differentiation (*p* = 0.005) and high p-TNM stage (*p* = 0.002) while BHLHE40 expression negatively correlated with cyclin D1 expression (*p* = 0.014), suggesting that BHLHE40 may act as a tumor suppressor in NSCLC [64]. A second study indicated that in squamous cell carcinoma (SCC) of the lung, but not in adenocarcinoma (ADC), low expression of BHLHE40 positively correlated with overall survival (OS) (p < 0.05), and favorable patient prognosis (p < 0.05) [83]. However, a third study which examined 115 tumor samples from patients with NSCLC (78 SCC and 37 ADC), showed that in both types, BHLHE40 was strongly expressed in the nuclei of normal bronchial epithelium and submucosal vessels, whereas it’s immunoreactivity was frequently reduced in cancer cells compared with adjacent normal bronchi [84]. In tumors that expressed nuclear BHLHE40, there was a strong and significant correlation with HIF1α and carbonic anhydrase-9 [84]. Together, these studies are consistent in suggesting BHLHE40 is reduced in NSCLC.

### Cancers where BHLHE40 expression is bimodal:

#### Esophageal carcinoma

In certain cancers, BHLHE40 is increased in tumors compared to normal tissue, but as the tumor progresses, its levels decrease. One such disease is esophageal squamous cell carcinoma (ESCC), where a study of 241 patients showed that BHLHE40 expression significantly increased in intraepithelial neoplasia (IEN) compared with normal precursor tissue [85]. However, thereafter, there was a significant decrease in BHLHE40 expression in ESCC compared with IEN [85]. In ESCC, expression of BHLHE40 positively correlated with senescence and with prolonged survival of ESCC patients after surgery. BHLHE40 also negatively correlated with age, tumor embolus, depth of invasion of ESCC, lymph metastasis status and pathological tumor lymph node metastasis stage (pTNMs) [85]. The authors suggested that BHLHE40 overexpression is a protective mechanism against ESCC progression. A second study using esophageal cancer cell lines showed that BHLHE40 overexpression promoted apoptosis as indicated by an increase in PARP cleavage [86]. Thus, while IEN is characterized by an increase in BHLHE40, progression to ESCC is characterized by its decrease.

#### Hepatocellular carcinoma

A study on hepatocellular carcinoma (HCC) demonstrated a distinction between active BHLHE40 in the nucleus and passive BHLHE40 in the cytoplasm. While BHLHE40 localized to the cytoplasm in hepatocytes of normal liver, HCC tissues showed high nuclear localization of BHLHE40, suggesting that nuclear BHLHE40 had a tumor promoting effect [87]. However, the frequency of nuclear BHLHE40 was higher in well differentiated, than in moderately, or poorly differentiated HCC [87], arguing that loss of BHLHE40 is associated with tumor progression. Thus, HCC truly represents the dichotomy of BHLHE40 – both as tumor promoter during initiation, but with loss of function in further progression.

#### Oral squamous cell carcinoma

In a study of 56 untreated patients, positive expression rate of BHLHE40 was significantly higher in oral squamous cell carcinoma (OSCC), than in normal oral mucosa (*n* = 20) (p <0.05) [88]. Within the OSCC cases, the expression of BHLHE40 was highest in patients who experienced recurrence within 1-year while it was lowest in those who experienced no recurrence in 3 years [89]. These results suggest that BHLHE40 functions as an oncogene in OSCC. However, the degree of BHLHE40 nuclear staining decreased with tumor progression from well-differentiated to moderately and poorly differentiated tumors [89], indicating a dichotomy of BHLHE40 function in tumor initiation and in tumor progression.

## FUNCTION OF BHLHE40 IN CANCER DEPENDS ON LOCALIZATION AND DOWNSTREAM EFFECTORS

The above narrative raises the question as to why BHLHE40 is upregulated in some cancers, while it is downregulated in others. BHLHE40 is a multi-functional protein that serves in different roles under different conditions. Cancer is caused by various factors, and BHLHE40’s role may be defined by the role of the protein in signaling related to these factors.

**Figure 4:**
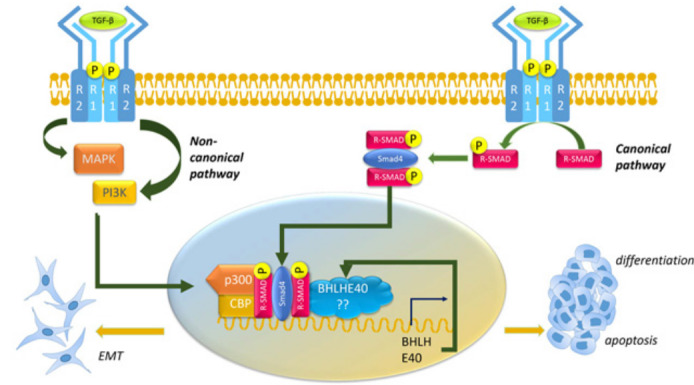
Schematic representation of BHLHE40 regulation by TGF-β. Ligands of the TGF-β superfamily bind to a type II receptor (R2), which recruits and phosphorylates a type I receptor (R1). Phosphorylated R1 in turn phosphorylates receptor-regulated Smads 2 and 3 (R-Smad) which bind the co-Smad Smad4. The R-Smad/Smad4 complex then translocates to the nucleus where they bind to and promote transcription of target genes, including BHLHE40. Newly synthesized BHLHE40 regulate additional transcriptional activity downstream of TGF-β and likely determines whether the cells undergo EMT and are pro-tumorigenic or undergo differentiation and apoptosis and are anti-tumorigenic. Factors that regulate this determination likely include co-factors such as CBP and p300 activated by non-canonical smad-independent pathways activated by TGF-β.

### Autoregulation of BHLHE40 in circadian rhythm

The best known functional role of BHLHE40 is in the regulation of the circadian rhythm [13, 40-42]. Multiple studies demonstrate that disruption of the circadian rhythm is a major cause of cancer initiation and/or progression. One mechanism by which BHLHE40 regulates tumorigenesis is via regulation of the circadian rhythm [61]. It suppresses transcription of genes important in the “clock genes”, such as Period 1 (Per1), by competing for E box sequences that the CLOCK:BMAL1 complex use to activate Per1 expression [13]. It was also shown to be involved in an auto feedback loop by CLOCK:BMAL1 that can regulate its expression [41]. CLOCK and BMAL1 heterodimerize and initiate the transcription of target genes that have E-box cis-regulatory sequences in their promoters, such as members of the Period genes and Cryptochrome (CRY) [90]. PER and CRY heterodimers in turn repress their own transcription by interacting with the CLOCK: BMAL1 complex [91-94]. Similarly, CLOCK:BMAL1 heterodimers activate the transcription of retinoic acid-related orphan nuclear receptors REV-ERBα and RORα [95], while the CRY:PER complex acts as a negative regulator and represses REV-ERBα and RORα expression once they have reached a critical concentration [95]. REV-ERBα and RORα compete to bind retinoic acid-related orphan receptor response elements (ROREs) in the promoter region of many genes that play a role in the circadian rhythm, such as BMAL1. Rorα was shown to activate the transcription of BMAL1 [96] while REV-ERBα repress it [95, 97]. Overexpression of BHLHE40 in both human and mouse cells cause a phase delay in circadian rhythms of the expression of E-box containing genes such as BHLHE40, BHLHE41, PER1 and REV-ERBα in the first cycle [42]. In contrast, deficiency in BHLHE40 advanced the circadian phase of these genes [42]. Additional studies indicate that BHLHE40 may play a role in resetting of the circadian clock independent of PER1 activation [98]. Activation of activin receptor-like kinase (ALK) [99], triggered by TGF-β, activin or alkali signals, reset the cellular clock independent of PER induction mediated by an immediate-early induction of BHLHE40 [98]. Many excellent reviews exploring the connection between the circadian rhythm and cancer has been written [100, 101], including from our group [102]; many of these have explored the tumor-suppressive role of BHLHE40 in the cross-talk between circadian rhythm and cancer [33]. Hence, here we will focus on non-circadian aspects of BHLHE40 cellular function in cancer.

### Differential effect of BHLHE40 in the nucleus and the cytoplasm

As noted above, BHLHE40 can be expressed in both the nucleus and the cytoplasm, and its functional role in cancer may vary accordingly (Figure 2B). BHLHE40 is upregulated in breast cancer compared to normal breast tissue [68-72] – and although the data presented do not refer to the localization of BHLHE40, accompanying immunohistochemical staining shows both nuclear as well as cytoplasmic staining in the tumor tissue [68, 71, 72]. The function of nuclear BHLHE40 was apparent from other studies, where nuclear BHLHE40 was up-regulated in MCF-7 estrogen receptor positive breast cancer cells upon paclitaxel treatment [103], suggesting that nuclear BHLHE40 prevent tumor progression. In contrast, cytoplasmic BHLHE40 bound to and stabilized cyclin E [104], thereby preventing its nuclear entry and inhibiting cell cycle progression, thus, an increase in cytoplasmic BHLHE40 is tumor suppressive. Therefore, in these cases, despite an upregulation, BHLHE40 may not play an oncogenic role.

Consistent with the above, nuclear BHLHE40 was persistently observed in almost all of the normal bronchial and alveolar tissue but in only 38% of NSCLC [84], suggesting a tumor suppressive role. In other cases, nuclear expression of BHLHE40 was seen in only a small fraction of cells in normal tissue; whereas in tumors, increased nuclear expression was observed in both epithelial and endothelial cells [105]. However, in the tumor, increased expression was mostly confined to areas of necrosis, while in morphologically viable cells, BHLHE40 was absent [105]. This likely indicates that increased nuclear BHLHE40 was specifically recruited to necrotic tissue and did not promote tumorigenesis. Another study showed that nuclear BHLHE40 suppressed cyclin D1 expression and cyclin D1 transcription [89]. Taken together, these studies indicate that an increase or decrease in BHLHE40 in cancer needs to be qualified by the localization of the protein.

### Downstream targets of BHLHE40 determine its role in tumor progression

Multiple BHLHE40 targets have been identified and are listed in Table 2, including STAT1 and STAT3. BHLHE40 homodimers bind to the E-box (5′-CACGTG-3′) sequence of the STAT1 promoter [106] with preference for elements preceded by T and/or followed by A residues, and is HDAC1-dependent [23]. This results in repression of targets of unphosphorylated STAT1, including antigen presenting genes and CASP1 [106]. Additionally, BHLHE40 binds to phosphorylated (active) STAT3α and -β isoforms at the HLH and C-terminal domains to activate STAT-dependent cis-elements [107], regulating transcription of the pro-apoptotic Fas gene [107]. Overexpression of BHLHE40 induced apoptosis whereas co-expression of STAT3β alleviated this effect [107]. Overall, these studies demonstrate that one way by which BHLHE40 suppresses tumor growth is by targeting JAK/STAT signaling.

BHLHE40 can also transactivate pro-tumorigenic factors. BHLHE40 upregulated the expression of *PIK3CA*, the gene that transcribes phosphatidylinositol 3-kinase (PI3K) [108], and elevated Akt phosphorylation, an oncogenic event [108, 89]. In turn, Akt phosphorylation increased BHLHE40 expression, thus activating a positive feedback loop [109]. Additionally, BHLHE40 negatively regulated 5’-adenosine monophosphate-activated protein kinase (AMPK), which regulates cell death by glucose depletion [110]. Paradoxically, metformin, which activates AMPK, induced the expression of BHLHE40 [111], suggesting the presence of a feedback loop, where metformin increases AMPK phosphorylation and BHLHE40 expression, but the increase in BHLHE40 then suppresses AMPK expression. Thus, BHLHE40 may have both tumor promoting and suppressive roles depending on the downstream targets that they affect.

## COOPERATIVITY OF BHLHE40 WITH OTHER FACTORS

The above illustrates the ability of BHLHE40 to affect both tumor promotion and suppression * but under different conditions*. Studies reveal that BHLHE40 expression is regulated by multiple pathways, which are either tumor promoting or suppressing. Depending on the pathway regulating its expression, and its cooperation with that pathway, BHLHE40 may be induced to either promote tumor progression or tumor regression.

**Figure 5:**
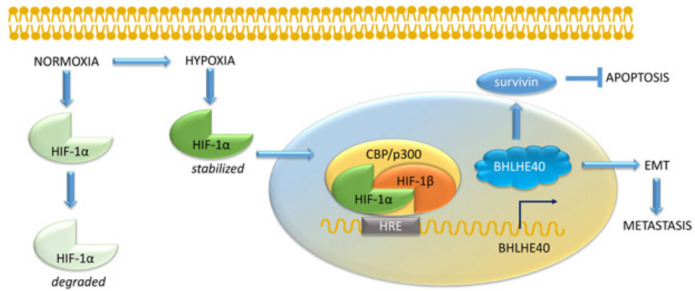
Regulation of BHLHE40 by hypoxia inducible factor 1α. The HIF complex is composed of a heterodimer of HIF-1α, which is induced in hypoxia, and HIF-1β, which is constitutively expressed. The complex binds to the hypoxia responsive elements (HRE) in the promoter region of target genes, including BHLHE40. In normoxia, HIF1-α is degraded, while hypoxia stabilizes HIF1-α, which then translocates to the nucleus to bind HIF-1β and co-regulators such as CBP/p300. In cooperation with the HIF complex, BHLHE40 protects cancer cells from apoptosis by upregulating survivin and caspase-3 activation, promoted EMT and induced metastasis.

### BHLHE40, p53-dependent DNA-damage repair and senescence

Multiple studies have shown that BHLHE40 is upregulated in response to stress stimuli, such as DNA damage, serum deprivation, hypoxia and various cytokines [11, 56, 112]. BHLHE40 is regulated by p53, a tumor suppressor that is activated by stress, including DNA damage [113] (Figure 3), and effectively limits cell proliferation that could lead to tumor initiation [114, 115]. BHLHE40 can be upregulated by DNA-damaging agents and ionizing radiation, which in turn enhanced p53 levels in a dose-dependent manner [57]. Interaction of BHLHE40 with p53 prevented its nuclear export supporting its role in p53-mediated responses [57] (Figure 3).

Senescent cells are metabolically active and can have tumor suppressive effects in the tumor microenvironment [116]. BHLHE40 is a component of the p53-dependent senescence pathway [117], and is upregulated by p53, leading to premature senescence [118]. Overexpression of BHLHE40 induced G1 arrest and promotes senescence in a p21-independent manner, whereas targeting endogenous BHLHE40 attenuated p53-mediated premature senescence [118]. The ability of BHLHE40 to induce cellular senescence was significantly reduced in p53-knockdown cells, indicating the necessity of p53-BHLHE40 cooperation in cellular senescence [118].

By inducing cellular senescence, however, BHLHE40 prevented p53-induced cell death. Under unstressed conditions, BHLHE40 is highly unstable and is targeted for proteasome-dependent degradation by the ubiquitin ligases SCF(βTrCP) and CK1 [119]. DNA damage induces BHLHE40 expression by p53, and is stabilized by the ubiquitin protease USP17, which extends its half-life [119]. However, BHLHE40 inhibits p53-dependent transcription of macrophage inhibitory cytokine-1 (MIC-1) by weakening promoter binding [120], whereas MIC-1 cooperates with BHLHE40 to prevent DNA damage-induced cell death [120]. BHLHE40 induced senescence was observed in esophageal carcinoma [85], NSCLC [121] and in OSCC [89] cells.

### BHLHE40 is a downstream effector of TGF-β/Smad in both tumor promotion and suppression

Transforming growth factor β (TGF-β) is a bi-functional growth factor that can either inhibit or stimulate cell proliferation in a context dependent manner [122]. TGF-β regulates a variety of functions in normal development, while the disruption of TGF-β signaling is associated with the onset of various types of cancer [122]. In addition to cell proliferation, TGF-β is also known to regulate cell differentiation and stemness, leading to effects on metastasis and immune modulation [122]. TGF-β ligands bind to receptors on the cell surface, which phosphorylates Receptor-phosphorylated Smad (R-Smad). Phosphorylated R-Smads bind to Smad 4 and the Smad complex then translocates to the nucleus where it binds target DNA sequences [24]. Smad-independent pathways that regulate TGF- β signaling have also been identified (Figure 4). BHLHE40 was identified as a target of TGF-β regulated Smad transcription in colorectal cancer, although it’s expression was independent of the growth inhibitory effects of TGF-β in these cells [24]. Significantly, TGF-β upregulated BHLHE40 but downregulated BHLHE41 [123] in a teratocarcinoma cell line, where it promoted chondrogenic differentiation [12], and in pancreatic cancer PANC-1 cells, where it regulated the subcellular localization of Smad3 phosphorylation and suppressed the expression of snail, claudin-4 and N-cadherin [81]. In these examples, therefore, BHLHE40 was involved in tumor suppression downstream of TGF-β.

In contrast, in mouse mammary carcinoma cell lines, *BHLHE40* promoted cell survival downstream of TGF-β activation [124]. BHLHE40 in other cell lines, mediated TGF-β induced morphological changes during epithelial-mesenchymal transition (EMT) leads to tumor migration and invasion [81]. Thus, BHLHE40 regulated tumorigenesis downstream of TGF- β in a context-dependent manner, upregulating tumor growth in some cases, downregulating it in others.

### BHLHE40 as a mediator of hypoxia-regulated tumor progression

Studies have shown that BHLHE40 is also regulated by hypoxia inducible factors (HIF). In hypoxic conditions, the HIF complex, composed of a heterodimer of HIF-1α and HIF-1β, binds to the hypoxia responsive elements (HRE) in the promoter region of target genes [125, 47, 126], including BHLHE40 [53]. While HIF-1β is expressed constitutively, HIF1-α is regulated by hypoxia. In normoxia, HIF1-α is unstable [126], while under hypoxic conditions, insufficient oxygen allows the stabilization of HIF1-α that translocate to the nucleus to bind HIF-1β (Figure 5). This allows the recruitment of co-activators that bind HREs to regulate transcription [127, 52, 128]. Overexpression of HIF-1α in 293T cells caused a 2-3 fold increase in BHLHE40 transcription [53], while BHLHE40 expression was shown to correlate with hypoxia and angiogenic markers such as HIF1α, angiogenin and VEGFD in breast cancer tissue [129-131].

In gastric cancers, hypoxia induced BHLHE40 expression while the HIF-1α protein inhibitor decreased the expression of BHLHE40 [54]. Additionally, BHLHE40 expression positively correlated with HIF-1α (P < 0. 01, r = 0.290) and Ki67 expression (P < 0. 01, r = 0.249) [67]. Under conditions of hypoxia, BHLHE40 protects gastric cancer cells from apoptosis by transcriptionally upregulating survivin [132]. Moreover, treatment with curcumin, which is known to protect from hypoxia, decreased HIF1α expression in gastric cancer cells, resulting in suppression of BHLHE40 expression [133]. Taken together, these studies indicate that in gastric cancer, an increase in BHLHE40 in tumor cells is indicative of hypoxia, which results in an increase of BHLHE40 expression driven by an increase in the HIF complex.

Other studies have noted that in hepatoma-derived cell lines, HIF-1α regulates the expression of BHLHE40 [55]. Further, overexpression of BHLHE40 antagonized apoptosis induced by 8-MOP by abolishing the decrease of survivin and the activation of caspase-3 [134]. Hypoxia-induced BHLHE40 promoted EMT and induced metastasis in HepG2 cells [135, 136]. Subsequent decrease in BHLHE40 with disease progression from well-differentiated to poorly differentiated tumors can also be explained by an increase in hypoxia, since in more extreme hypoxia there may be an induction of cell death [105]; causing BHLHE40 to be depleted [87, 89]. Therefore, hypoxia-induced expression of BHLHE40 is mostly pro-tumorigenic.

### Effect of BHLHE40 in NOTCH signaling in thyroid cancer

**Figure 6:**
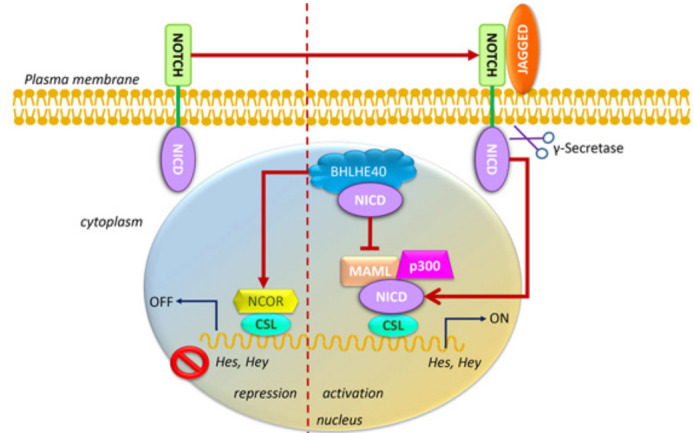
Schematic representation of the regulation of BHLHE40 by Notch. Following ligand binding to the Notch receptor that is localized into the plasma membrane, endocytosis of the ligand-receptor complex is initiated when the γ-secretase cleaves the Notch extracellular domain away from the transmembrane domain, NICD. CSL is a DNA-binding adaptor molecule that can interact with various repressors, such as NCOR, or with activators, such as NICD, and others. NICD translocates to the nucleus and can bind with CSL. This recruits the adaptor protein Mastermind-like (MAML), which in turn recruits the histone acetyltransferase p300. This allows transcription regulation of Notch-mediated target genes, such as Hes and Hey. BHLHE40 binding to NICD determines whether CSL mediated transcription is turned on or off.

Not surprisingly, BHLHE40 has a very similar effects in Notch signaling in cancer, which induced aggressive regeneration of tumors in thyroid cancer models (Figure 6). Studies showed that BHLHE40 sustained progression of thyroid cancer by promoting cell growth and invasiveness [77]. Aberrant signaling in thyroid cancer cells ensured that BHLHE40 cooperated with Notch signaling to promote target gene transcription and tumor aggression [77]. In both satellite cells and in thyroid cancer, BHLHE40 increased the expression of the Notch ligand Jagged, however, Jagged in satellite cells attenuated Notch signaling whereas in thyroid cancer, it promoted Notch signaling [77, 46]. The cause for this difference, or indeed whether it is due cancer vs non-cancer or a general difference between satellite cells and thyroid epithelial cells, is not yet known. Regardless, these reports indicate that in collaboration with Notch, BHLHE40 promoted cancer.

## CONSEQUENCES OF BHLHE40 ACTIVATION

### Immunomodulatory effects of BHLHE40 in cancer

We showed above that BHLHE40 may suppress the growth of colorectal carcinoma by expressing BHLHE40^+^ T_H_1-like immunoregulatory cells enriched in tumors with microsatellite-insatiability [80]. These cells are known to be responsive to immune-checkpoint blockade. Anti-PD-L1 inhibitors such as avelumab are now used in many cancers such as Merkel cell carcinoma (MCC) [137]. BHLHE40 expression was critical for tumor-infiltrating lymphocytes (TIL) reinvigoration following anti-PD-L1 blockade [138]. On the flip side, PD-1 signaling inhibited BHLHE40 expression in TIL [138]. Peritoneal macrophages are known to play immunological functions in abdominal cancers [139]. BHLHE40 is highly expressed in large peritoneal macrophages (LPM) [140]. Loss of BHLHE40 expression prevented LPM expansion [140]. These papers point to a strong immunomodulatory role for BHLHE40 in cancer.

### Dual effect of BHLHE40 on apoptosis

A common theme that has emerged in the review of the literature is that BHLHE40 promotes cancer when it has anti-apoptotic functions while it acts as a tumor suppressor when it promotes apoptosis. In patients with recurrent glioblastoma, BHLHE40 expression correlated negatively with apoptosis [74]. In colon cancer, BHLHE40 overexpression impeded serum deprivation-induced apoptosis and selectively inhibited the activation of procaspases related to the intrinsic death pathway (precaspase 3, 7, 9), without affecting the extrinsic death pathway [79]. On the other hand, a study using esophageal cancer cell lines showed that BHLHE40 overexpression promoted apoptosis as indicated by an increase in PARP cleavage [86]. This contrast can be explained by investigation of the downstream targets of BHLHE40 in cancers where it either promotes or inhibits apoptosis. BHLHE40 can promote apoptosis by binding to phosphorylated (active) STAT3α and -β isoforms at the HLH and C-terminal domains to activate STAT-dependent cis-elements [107], thereby regulating transcription of the pro-apoptotic Fas gene [107]. In tumors where BHLHE40 has this effect, overexpression of BHLHE40 induced apoptosis [107]. In contrast, under conditions of hypoxia, BHLHE40 protects gastric cancer cells from apoptosis by transcriptionally upregulating survivin [132]. Under these conditions, overexpression of BHLHE40 antagonized apoptosis induced by 8-MOP by abolishing the decrease of survivin and the activation of caspase-3 [134]. In breast cancer cells, clusterin (CLU) was identified as a novel target gene of BHLHE40 and suppressed DNA damage-induced cell death [141]. BHLHE40 was found to be a strong regulator of the PI3K/Akt/mTOR pathway. BHLHE40 upregulated the expression of *PIK3CA*, the gene that transcribes phosphatidylinositol 3-kinase (PI3K) [108], and elevated Akt phosphorylation, a downstream target of PI3K that is a known regulator of cell survival [108, 89]. Thus, BHLHE40-mediated tumor suppression can be traced to inhibition of oncogenic factors such as STAT1, whereas BHLHE40-mediated promotion of tumor progression may be traced to the activation of the PI3K/Akt/mTOR pathway and the upregulation of pro-survival factors such as survivin and clusterin.

## SUMMARY AND CONCLUSIONS

In this article, we discuss the role of BHLHE40 in oncogenesis, since it is overexpressed in some cancers and suppressed in others. We conclude that BHLHE40 overexpression does not always indicate increased activity, such as in breast cancer, where it is expressed in the cytoplasm, suppressing cell growth by stabilizing cyclin E. In the nucleus, BHLHE40 either suppressed tumors by inhibiting the expression of STAT1 or promoted tumor progression by activating the PI3K/Akt/mTOR pathway or by repressing AMPK. Further, we show that BHLHE40 selects its targets by cooperation with other transcription factors that regulate the expression, and the function, of BHLHE40, such as HIF1α in gastric cancer and in HCC. This explains the pro-tumorigenic role of BHLHE40 in these diseases. Its interaction with the p53 in lung cancer and esophageal carcinoma, induces senescence and suppresses tumor growth. Thus, BHLHE40 is a multi-functional gene that mediates the promotion or suppression of cancer in a context dependent manner. Future studies should compare the transcriptional targets of BHLHE40 in tumors where it is tumor suppressive and compare them to tumors where it is oncogenic in order to determine the mode of action of this transcription factor in cancer.
